# Supplementary material for: Hybrid computational modeling highlights reverse warburg effect in breast cancer-associated fibroblasts
Source: Comput Struct Biotechnol J. 2023 Aug 20;21:4196–206. doi: 10.1016/j.csbj.2023.08.015 (PMC10495551; doi:10.1016/j.csbj.2023.08.015)
Supplement: Supplementary file 1 — Supplementary material [file mmc1.pdf]

Table S1. Breast CAFs-specific initial conditions generated from data-driven differential expression analysis (DEA) or manual curation of peer-reviewed literature (PubMed IDs are provided).

Component	Function	Initial value	Source
ACTA1	Internal node	0	DEA
ACTA1_rna	Internal node	0	DEA
ACTG2	Internal node	0	DEA
ACTG2_rna	Internal node	0	DEA
AKT1S1_phosphorylated	Internal node	1	DEA
AREG_Cytosol	Internal node	0	DEA
AREG_extracellular	Input node	0	DEA
AREG_rna	Internal node	0	DEA
AREG_Secreted_compartment	Internal node	0	DEA
ARHGEF7	Internal node	1	DEA
BMP4	Internal node	1	DEA
BMP4_rna	Internal node	1	DEA
CALML3	Internal node	0	DEA
CASQ2	Input node	0	DEA
CAV3	Internal node	0	DEA
CAV3_rna	Internal node	0	DEA
CCL11	Internal node	1	DEA
CCL11_rna	Internal node	1	DEA
CCL8	Internal node	0	DEA
CCL8_rna	Internal node	0	DEA
CFL1	Input node	1	22954256
Collagens_extracellular	Input node	1	31521169
CTGF_extracellular	Input node	1	34108441
CXCL1	Internal node	1	DEA
CXCL1_rna	Internal node	1	DEA
CXCL12_extracellular	Input node	1	DEA
CXCL12_rna	Internal node	1	DEA
CXCL12_Secreted_compartment	Internal node	1	DEA
CXCL12CXCR4_complex	Internal node	1	DEA
CXCL2	Internal node	1	DEA
CXCL2_rna	Internal node	1	DEA
EGF_extracellular	Input node	1	35267539
EGFR	Internal node	1	DEA
EGFR_complex	Internal node	1	DEA
EGFR_rna	Internal node	1	DEA
EMILIN1	Internal node	1	DEA
FAP	Internal node	1	DEA
FAP_rna	Internal node	1	DEA
FGF1	Input node	1	33568624
FGF2_extracellular	Input node	1	32557854
FGF3	Input node	1	35267539
FGF4	Input node	1	33081025
FGF7	Internal node	1	DEA
FGF7_rna	Internal node	1	DEA
FGFRFGF1_complex	Internal node	1	DEA
FGFRFGF2_complex	Internal node	1	DEA
FGFRFGF3_complex	Internal node	1	DEA
FGFRFGF4_complex	Internal node	1	DEA
FN_extracellular	Input node	1	35481621
GAST	Input node	1	28560291
GASTCCKBR_complex	Internal node	0	DEA
GLI2	Internal node	1	DEA
GLI2_rna	Internal node	1	DEA
GLNS	Input node	1	27829138
M_glc_D_e_simple_molecule	Input node	1	DEA
HGF_extracellular	Input node	1	22282252
HIF1A	Internal node	1	DEA
HRC	Input node	0	DEA
IFNG	Input node	1	33005420
IGF1_extracellular	Input node	1	DEA
IGF1_rna	Internal node	1	DEA
IGF1_Secreted_compartment	Internal node	1	DEA
IGF2_extracellular	Input node	1	DEA
IGF2_rna	Internal node	1	DEA
IGF2_Secreted_compartment	Internal node	1	DEA
IGFBP3	Input node	1	DEA
IGFBP4	Input node	1	19536088
IHH	Input node	1	35224148
IL12	Input node	0	DEA
IL18	Input node	0	31231372
IL1A	Input node	1	31231372
IL1B_extracellular	Input node	1	31231372
IL1R_complex_Cytosol	Internal node	0	DEA
IL1R_complex_Cytosol_2	Internal node	0	DEA
IL6_extracellular	Input node	1	35267539
IRS2_phosphorylated	Internal node	1	DEA
ITGA11	Internal node	1	DEA
ITGA11_rna	Internal node	1	DEA
ITGA11ITGB1_complex_Cytosol	Internal node	1	DEA
ITGA11ITGB1_complex_Cytosol_active	Internal node	1	DEA
ITGA1ITGB1_complex_Cytosol	Internal node	0	DEA
ITGA1ITGB1_complex_Cytosol_active	Internal node	0	DEA
ITGAVITGB6_complex_Cytosol	Internal node	0	DEA
ITGAVITGB6_complex_Cytosol_active	Internal node	0	DEA
Large_Latent_Complex_complex_extracellular	Input node	1	DEA
Large_Latent_Complex_complex_Secreted_compartment	Internal node	1	DEA
LGALS1	Input node	1	24229053
LIF_extracellular	Input node	1	34947829
LIMK1_phosphorylated	Internal node	1	DEA
LOX	Internal node	1	DEA
LOX_rna	Internal node	1	DEA
LPA_simple_molecule	Input node	0	DEA
MAP3K7TAB_complex	Internal node	1	DEA
MIF	Input node	0	24939415
MIR101_antisense_rna	Input node	0	28289080
MIR141_antisense_rna	Input node	0	28289080
MIR155_antisense_rna	Input node	1	23171795
MIR200B_antisense_rna	Input node	0	28289080
MIR205_antisense_rna	Input node	0	28289080
MIR211_antisense_rna	Input node	1	31702390
MIR214_antisense_rna	Input node	0	23171795
MIR221_antisense_rna	Input node	1	28289080
MIR31_antisense_rna	Internal node	1	28289080
MMP13	Internal node	1	DEA
MMP13_rna	Internal node	1	DEA
MMP14	Internal node	1	DEA
MMP14_rna	Internal node	1	DEA
MMP2	Internal node	1	DEA
MYLK_phosphorylated	Internal node	0	DEA
NDUFA4L2	Internal node	0	DEA
NOX4	Internal node	1	DEA
NOX4_rna	Internal node	1	DEA
OSM	Input node	1	35192545
PDGF_extracellular	Input node	1	34272173
PDGFPDGFRA_complex	Internal node	1	DEA
PDGFRA	Internal node	1	DEA
PDGFRA_rna	Internal node	1	DEA
PGE2_simple_molecule_extracellular	Input node	1	33271839
phospholipid_simple_molecule	Input node	1	
PI45P2_simple_molecule	Input node	1	34108441
PLAU	Internal node	1	DEA
PLAU_rna	Internal node	1	DEA
PLG	Input node	1	33921488
POSTN_extracellular	Input node	1	35267539
PPBP	Internal node	0	DEA
PPBP_rna	Internal node	0	DEA
proPLAU_extracellular	Input node	1	24229053
PTCH2_rna	Internal node	1	DEA
PTGS2	Internal node	1	DEA
PTGS2_rna	Internal node	1	DEA
PTPN6	Input node	0	DEA
RYR2TRDNASPH_complex	Input node	0	DEA
RYR2TRDNASPHHRCCASQ2_complex	Internal node	0	DEA
SEPTINE4	Internal node	0	DEA
SERPINE1	Internal node	1	DEA
SERPINE1_rna	Internal node	1	DEA
SHH	Input node	1	28496132
SMOPTCH_complex	Internal node	1	DEA
SMOX	Internal node	1	DEA
TGFB3_Cytosol	Internal node	1	DEA
TGFB3_extracellular	Internal node	1	DEA
TGFB3_rna	Internal node	1	DEA
TNF	Input node	0	DEA
VTN	Input node	1	33211735
WAS_phosphorylated	Internal node	0	DEA
WNT7	Input node	1	34108441
WWTR1	Input	1	DEA
