# Supplementary material for: Hybrid computational modeling highlights reverse warburg effect in breast cancer-associated fibroblasts
Source: Comput Struct Biotechnol J. 2023 Aug 20;21:4196–206. doi: 10.1016/j.csbj.2023.08.015 (PMC10495551; doi:10.1016/j.csbj.2023.08.015)
Supplement: Supplementary file 2 — Supplementary material [file mmc2.pdf]

Table S2. Biological scenarios identified from breast cancer-specific peer-reviewed literature used for generic validation of the breast CAF-model's behavior in the Cell Collective platform. Biological experimental conditions were used as the model's inputs and its outputs were compared to the biological outcome to confirm or refute a scenario's validation.

Experimental observation	Reference	CAF model input	CAF model output	Coherence
A loss of Caveolin induces the metabolic reprogramming of breast CAFs by increasing mitochondrial dysfunction.	22874531	CAV-3 OFF/ON	CI, CIV OFF/ON	**YES**
Breast CAFs recombinantly expressing TGF-β show upregulation of myofibroblast markers β-actin.		TGFB3 OFF/ON	Actin cytoskeletal OFF/ON	**YES**
Breast CAFs recombinantly expressing TGF-β show a loss of Cav-1/3 expression.		TGFB3 OFF/ON	CAV3_rna ON/OFF	**YES**
Tumors derived from TGF-β ligand overexpressing breast CAFs display increased extracellular matrix deposition and increased secretion of: (a) Collagens, (b) Tenascin C.		TGFB3 ON/OFF	COLLAGENS OFF/OFF	**NO** (Missing mechanistic information)
			TNC OFF/OFF	**NO** (Missing mechanistic information)
Breast CAFs promote tumor growth, in an angiogenesis-independent manner when overexpressing TGF-β3.		TGFB3 OFF/ON	Tumor growth OFF/ON	**YES**
Upregulation of miR-221 in breast CAFs affects growth and migration by CTGF signaling pathway.	29716623	miR-221 ON	Tumor growth OFF/OFF	**NO** (Missing mechanistic information)
Downregulation of miR-320 in breast CAFs promotes tumor angiogenesis.	22179046	miR-320 OFF/ON	Angiogenesis OFF/ON	**YES**
miR-155 promotes proliferation of human breast CAFs.	24152184	miR-15 OFF/ON	Fibroblast proliferation OFF/ON	**YES**
Blockage of MAPK/p38 pathway diminished IL-32-induced tumor growth.	30391782	MAPK, p38 OFF/ON	Tumor growth OFF/ON	**YES**
Enhanced WNT expression in breast CAFs contributes to invasion and migration of breast cancer cells.	32366558	WNT7 OFF/ON	Migration into tumor OFF/ON	**YES**
In breast CAFs, IL-1β promotes cell invasion through IL-1R.	27072893	IL-1β OFF	Tumor invasion OFF/OFF	**NO** (Missing mechanistic information)

CAFs' PDGF signaling has an active role in breast tumor progression.	29380207	PDGF OFF/ON	Tumor growth, migration OFF/ON	YES
In breast CAFs, CXCL12 promotes cell invasion through TGF-β pathway.	31964880	CXCL12 OFF/ON	Tumor invasion OFF/ON	YES
In breast CAFs, FGF2 promotes cancer growth and progression.	32557854	FGF2 OFF/ON	Tumor growth, growth factors OFF/ON	YES
CXCL1 is considered as a pro-inflammatory gene signature of breast CAFs.	23831470	CXCL1 OFF/ON	Cytokine production, immune system modulation OFF/ON	YES
Breast CAFs promote tumor growth through secretion of HGF.	22282252	HGF OFF/ON	Tumor growth OFF/ON	YES
miR-21 and miR-200B target TGF-β signaling and impact tumor progression and promotion in breast CAFs.	27153853	miR-21 OFF		**NO** (Missing mechanistic information)
		miR-200B OFF		**NO** (Missing mechanistic information)
Myeloid-derived OSM reprograms breast CAFs to a more tumorigenic phenotype by eliciting the secretion of VEGF.	35192545	OSM OFF/ON	VEGF OFF/ON	YES
OSM promoted tumor growth through breast CAFs.			Tumor growth OFF/ON	YES
OSM induced the expression of classical CAF markers such as FAP, POSTN, VEGF, and IL6 in breast CAFs.			FAP, POSTN, VEGF, IL6 OFF/ON	YES
OSM induced signatures related to fibroblast activation and JAK/STAT3 signaling, in agreement with increased STAT3 phosphorylation by OSM in breast CAFs.			STAAT3 OFF/OFF	**NO** (Missing mechanistic information)
Plasmin expression affects tumor cell invasion.	10190278	Plasmin OFF/ON	Matrix degradation, matrix effects OFF/ON	YES
Plasmin expression is required for activation of EMT in breast cancer.	19546228	Plasmin ON/OFF	Matrix effect OFF/OFF	**NO** (Missing mechanistic information)
TGF-β promotes CXCL5 secretion in breast CAFs.	32237072	TGF-β ON/OFF	CXCL5 ON/OFF	YES
Lactate production in breast CAFs promotes breast cancer tumor growth.	22129993	Lactate OFF/ON	Tumor growth OFF/ON	YES
TFAM-deficient breast CAFs showed evidence of mitochondrial dysfunction.		TFAM OFF/ON	CI, CV OFF/ON	YES
SIRT3 in breast CAFs was found to:<br>(a) Suppress HIF-1α and its target genes;	22589271; 22749020	SIRT3 OFF/ON	HIF1A OFF/OFF	**NO** (Missing mechanistic

(b) Suppress tumor growth and proliferation;<br>(c) Suppress ROS production.				information)
			Tumor growth, tumor fibroblast proliferation OFF/ON	**YES**
			ROS production OFF/ON	**YES**
Breast CAFs promote tumor growth and angiogenesis through elevated CXCL12 secretion.	15882617	CXCL12 OFF/ON	Tumor growth, Angiogenesis OFF/ON	**YES**
LOX family members are considered as ECM-modifying enzymes in breast CAFs by remodeling the extracellular matrix.	33037194	LOX OFF/ON	ECM regulation phenotype and matrix effects OFF/ON	**YES**
Downregulation of MiR-205 in breast CAFs promotes VEGF-independent angiogenesis through activation of IL-11/IL-15 signaling by YAP1.	29109792	MiR-205 OFF		**NO**<br>(Co-activator needed)
In breast cancer, HIF-1α transcriptionally upregulates glycolytic enzymes and lactate production.	28623342	HIF1A OFF/ON	Glycolytic enzymes, lactate OFF/ON	**YES**
Lactate generated by hypoxic breast CAFs promotes cell invasion.	30799198	Lactate OFF/ON	Tumor invasion OFF/ON	**YES**
Inhibiting NF-κB signaling in fibroblasts was shown to reduce inflammatory cytokine secretion.	28378188	NFkB OFF/ON	Cytokine production phenotype OFF/ON	**YES**
Ets2 inactivation through depletion of Pten in breast CAFs was sufficient to decrease tumor growth and progression.	19847259	PTEN OFF		**NO**<br>(Co-activator needed)
SERPINE1 promotes cellular invasion in breast CAFs.	33000256	SERPINE1 OFF/ON	Tumor invasion OFF/ON	**YES**
CASQ2 overexpression accelerated tumorigenesis, induced collagen structure remodeling, and increased distant metastasis.	34743414	CASQ2 OFF/ON	Collagens OFF/OFF	**NO** (Missing mechanistic information)
Hypoxic breast CAFs led to sustained elevation of HIF-1α.	32492417	Hypoxia OFF/ON	HIF1A OFF/ON	**YES**
