# Supplementary material for: Hybrid computational modeling highlights reverse warburg effect in breast cancer-associated fibroblasts
Source: Comput Struct Biotechnol J. 2023 Aug 20;21:4196–206. doi: 10.1016/j.csbj.2023.08.015 (PMC10495551; doi:10.1016/j.csbj.2023.08.015)
Supplement: Supplementary file 4 — Supplementary material [file mmc4.pdf]

Table S4. Metabolic enzymes with projected maximal regulatory trap-space equal to 0 in breast CAF-specific initial conditions and their associated catalyzed reaction constrained to 0 in MitoCore.

Enzyme	Complete Name	Metabolic subsystem	Catalyzed reaction	Detailed reaction
GLUNm	Mitochondrial Glutaminase	Glutamine degradation	R_GLUNm	L-Glutamine[m] + H_2O[m] + NAD^+[m] → L-Glutamate[m] + NH_4[m]
CI_MitoCore	NADH Dehydrogenase	Electron transport chain	R_CI_MitoCore	H^+[m] + NADH[m] + Ubiquinone[m] ⇌ NAD^+[m] + H^+[m] + Ubiquinol[m]
HMGCOASim	Hydroxymethylglutaryl Coenzyme A Synthase	Ketogenesis	R_ HMGCOASim	Acetyl-CoaA[m] + Acetoacetyl-CoA[m] + H_2O[m] ⇌ 3-Hydroxy-3-methylglutaryl-CoA[m] + CoA[m]
CIII_MitoCore	Cytochrome C Reductase	Electron transport chain	R_CIII_MitoCore	H^+[m] + Ubiquinol[m] + Ferricytochrome C[m] ⇌ H^+[m] + Ubiquinone[m] + Ferrocytochrome C[m]
CIV_MitoCore	Cytochrome C Oxidase	Electron transport chain	R_CIV_MitoCore	H^+[m] + O_2[m] + Ferrocytochrome C[m] → H^+[m] + H_2O[m] + Ferricytochrome C[m]
ICDHxm	Isocitrate Dehydrogenase	Tricarboxylic acid cycle	R_ICDHxm	Isocitrate[m] + NAD^+[m] → 2-Oxoglutarate[m] + CO_2[m] + NADH[m]
PDHm	Pyruvate Dehydrogenase	Tricarboxylic acid cycle	R_PDHm	Pyruvate[m] + CoA[m] + NAD^+[m] → Acetyl-CoA[m] + CO_2[m] + NADH[m] + H^+[m]
