# Supplementary material for: Hybrid computational modeling highlights reverse warburg effect in breast cancer-associated fibroblasts
Source: Comput Struct Biotechnol J. 2023 Aug 20;21:4196–206. doi: 10.1016/j.csbj.2023.08.015 (PMC10495551; doi:10.1016/j.csbj.2023.08.015)
Supplement: Supplementary file 5 — Supplementary material [file mmc5.pdf]

Table S5. Metabolic enzymes with projected maximal regulatory trap-space equal to 0 in breast CAF-specific initial conditions and their associated catalyzed reaction constrained to 0 in MitoCore.

Metabolite	Complete name	Metabolic subsystem	Producing reaction	Detailed reaction
M_gln_L_c	L-Glutamine [cytoplasmic]	Glutamine synthesis	R_GLNS	ATP [c] + L-Glutamate[c] + NH_4[c] → **L-Glutamine [c]** + ADP[c] + Orthophosphate[c]
		L-Glutamine transport	R_r2525	L-Glutamine[e] ⇌ **L-Glutamine [c]**
M_hmgcoa_m	3-Hydroxy-3-methylglutaryl-CoA [mitochondrial]	Leucine degradation	R_MGCHrm	3-Methylglutaconyl-CoA[m] + H_2O[m] ⇌ **3-Hydroxy-3-methylglutaryl-CoA**[m]
		Ketogenesis	R_ HMGCOASim	Acetyl-CoaA[m] + Acetoacetyl-CoA[m] + H_2O[m] ⇌ **3-Hydroxy-3-methylglutaryl-CoA**[m] + CoA[m]
M_accoa_m	Acetyl-CoA [mitochondrial]	Isoleucine degradation	R_ACACT10m	CoA[m] + 2-Methylacetoacetyl-CoA[m] → Propanoyl-CoA[m] + **Acetyl-CoA[m]**
		FA and ketone body metabolism, ketogenesis	R_ACACT1rm	**Acetyl-CoA[m]** ⇌ CoA[m] + Acetoacetyl-CoA[m]
		TCA cycle periphery	R_ACITLm_MitoCore	ATP[m] + Citrate[m] + CoA[m] → ADP[m] + Orthophosphate[m] + **Acetyl-CoA[m]** + Oxaloacetate[m]
		Alcohol metabolism	R_ACSm	ATP[m] + Acetate[m] + CoA[m] → AMP[m] + Diphosphate[m] + **Acetyl-CoA[m]**
		Ketogenesis / Leucine degradation	R_HMGLm	3-Hydroxy-3-methylglutaryl-CoA[m] → **Acetyl-CoA[m]** + Acetoacetate[m]
		Beta-alanine degradation	R_MMSAD3m	3-Oxopropanoate[m] + CoA[m] + NAD^+[m] → **Acetyl-CoA[m]** + CO_2[m] + NADH[m] + H^+[m]
		Beta-alanine degradation	R_MMSAD3m2_MitoCore	3-Oxopropanoate[m] + CoA[m] + $NADP^+$[m] → **Acetyl-CoA[m]** + CO_2[m] + NADPH[m] + H^+[m]
		Fatty acid metabolism	R_MTPC14_MitoCore	Trans-Hexadec-2-enoyl-CoA[m] + H_2O[m] + NAD^+[m] + CoA[m] ⇌ Lauroyl-CoA[m] + NADH[m] + **Acetyl-CoA[m]**

			R_MTPC16_MitoCore	Trans-Hexadec-2-enoyl-CoA[m] + H_2O[m] + NAD^+[m] + CoA[m] ⇌ Tetradecanoyl-CoA[m] + NADH[m] + **Acetyl-CoA[m]**
			R_r0287	CoA[m] + 3-Oxohexanoyl-CoA[m] ⇌ **Acetyl-CoA[m]** + Butanoyl-CoA[m]
			R_r0634	Octanoyl-CoA[m] + **Acetyl-CoA[m]** ⇌ CoA[m] + 3-Oxodecanoyl-CoA[m]
			R_r0724	Decanoyl-CoA[m] + **Acetyl-CoA[m]** ⇌ CoA[m] + 3-Oxododecanoyl-CoA[m]
			R_r0732	CoA[m] + 3-Oxooctanoyl-CoA[m] ⇌ Hexanoyl-CoA[m] + **Acetyl-CoA[m]**
		Glycolysis, gluconeogenesis	R_PDHm	Pyruvate[c] + CoA[c] + NAD^+[c] → **Acetyl-CoA[c]** + CO_2[c] + NADH[c] + H^+[c]
M_akg_m	2-Oxoglutarate [mitochondrial]	Lysine degradation	R_2AMADPTmC_MitoCore	**2-Oxoglutarate[m]** + L-Aminoadipate[m] → 2-Oxoglutarate[c] + L-Aminoadipate[c]
		Tryptophan Metabolism	R_2OXOADPTmC_MitoCore	**2-Oxoglutarate[m]** + 2-Oxoadipate[c] → 2-Oxoglutarate[c] + 2-Oxoadipate[m]
		Malate aspartate shuttle	R_ASPTAm	L-Aspartate[m] + **2-Oxoglutarate[m]** ⇌ Oxaloacetate[m] + L-Glutamate[m]
		Glutamate degradation and synthesis	R_GLUDxm	L-Glutamate[m] + NAD^+[m] + H_2O[m] → **2-Oxoglutarate[m]** + NH_4[m] + NADH[m] + H^+[m]
			R_GLUDym	L-Glutamate[m] + $NADP^+$[m] + H_2O[m] → **2-Oxoglutarate[m]** + NH_4[m] + NADPH[m] + H^+[m]
		Tricarboxylic acid cycle	R_ICDHxm	Isocitrate[m] + NAD^+[m] → **2-Oxoglutarate[m]** + CO_2[m] + NADH[m]
			R_ICDHyrm	Isocitrate[m] + $NADP^+$[m] ⇌ **2-Oxoglutarate[m]** + CO_2[m] + NADPH[m]
M_cit_m	Citrate [mitochondrial]	Mitochondrial transporters	R_CITtamB	Citrate[c] + L-Malate[m] → **Citrate[m]** + L-Malate[c]
			R_CITtbm	Citrate[c] + Phosphoenolpyruvate[m] → **Citrate[m]** + Phosphoenolpyruvate[c]
			R_r0917	Citrate[c] + Isocitrate[m] → **Citrate[m]** + Isocitrate[c]
			R_r0917b_MitoCore	Citrate[c] + Isocitrate[m] → **Citrate[m]** + Isocitrate[c]

		Tricarboxylic acid cycle	R_CSm	Acetyl-CoA[m] + H_2O[m] + Oxaloacetate[m] → **Citrate[m]** + CoA[m]
M_fum_m	Fumarate [mitochondrial]	Electron transport chain	R_CII_MitoCore	FAD[m] + Succinate[m] ⇌ **Fumarate[m]** + $FADH_2$[m]
		Mitochondrial transporters	R_FUMtmB_Mitocore	Fumarate[c] + Pi[m] → **Fumarate[m]** + Pi[c]
M_icit_m	Isocitrate [mitochondrial]	Tricarboxylic acid cycle	R_ACONTm	Citrate[m] ⇌ **Isocitrate[m]**
M_mal_L_m	L-Malate [mitochondrial]	Malate aspartate shuttle	R_AKGMALtm	L-Malate[c] + 2-Oxoglutarate[m] ⇌ **L-Malate[m]** + 2-Oxoglutarate[c]
		Mitochondrial transporters	R_MALSO3tm	L-Malate[c] + Sulfite[m] ⇌ **L-Malate[m]** + Sulfite[c]
			R_MALSO4tm	L-Malate[c] + Sulfate[m] ⇌ **L-Malate[m]** + Sulfate[c]
			R_MALTSULtm	L-Malate[c] + Thiosulfate[m] ⇌ **L-Malate[m]** + Thiosulfate[c]
			R_r0913	L-Malate[c] + Isocitrate[m] → **L-Malate[m]** + Isocitrate[c]
		Tricarboxylic acid cycle	R_FUMm	Fumarate[m] + H_2O[m] ⇌ **L-Malate[m]**
M_oaa_m	Oxaloacetate [mitochondrial]	Tricarboxylic acid cycle periphery	R_ACITLm_MitoCore	ATP[m] + Citrate[m] + CoA[m] → ADP[m] + Orthophosphate[m] + Acetyl-CoA[m] + **Oxaloacetate[m]**
			R_MDHm	L-Malate[m] + NAD^+[m] ⇌ **Oxaloacetate[m]** + NADH[m] + H^+[m]
			R_PCm	ATP[m] + Pyruvate[m] + $HCO3^-$[m] → ADP[m] + Orthophosphate[m] + **Oxaloacetate[m]**
M_gln_L_m	L-Glutamine [mitochondrial]	Mitochondrial transporters	R_GLNtm	L-Glutamine[c] ⇌ **L-Glutamine**[m]
M_succ_m	Succinate [mitochondrial]	Ketone bodies degradation	R_OCOAT1m	Succinyl-CoA[m] + Acetoacetate[m] ⇌ **Succinate[m]** + Acetoacetyl-CoA[m]
		Mitochondrial transporters	R_SUCCt2m	Succinate[c] + Pi[m] ⇌ **Succinate[m]** + Pi[c]
			R_SUCCt3m_MitoCore	Succinate[c] + L-Malate[m] ⇌ **Succinate[m]** + L-Malate[c]

			R_r0829	Succinate[c] + Sulfate[m] ⇌ **Succinate[m]** + Sulfate[c]
			R_r0830	Succinate[c] + Sulfite[m] ⇌ **Succinate[m]** + Sulfite[c]
			R_r0830B_MitoCore	Succinate[c] + Thiosulfate[m] ⇌ **Succinate[m]** + Thiosulfate[c]
		Tricarboxylic acid cycle	R_SUCOAS1m	GDP[m] + Orthophosphate[m] + Succinyl-CoA[m] ⇌ GTP[m] + **Succinate[m]** + CoA[m]
			R_SUCOASm	GDP[m] + Succinyl-CoA[m] ⇌ GTP[m] + **Succinate[m]**
		GABA shunt	R_r0178	Succinate semialdehyde[m] + NAD^+[m] + H_2O[m] → **Succinate[m]** + NADH[m] + H^+[m]
M_succoa_m	Succinyl-CoA [mitochondrial]	Tricarboxylic acid cycle	R_AKGDm	2-Oxoglutarate[m] + CoA[m] + NAD^+[m] → **Succinyl-CoA[m]** + CO_2[m] + NADH[m] + H^+[m]
		Propanoate metabolism	R_MMMm	(R)-Methylmalonyl-CoA[m] ⇌ **Succinyl-CoA[m]**
M_acac_m	Acetoacetic acid [mitochondrial]	Mitochondrial transporters	R_ACACt2mB_MitoCore	Acetoacetic acid[c] + H^+[c] ⇌ **Acetoacetic acid[m]** + H^+[m]
		Ketone bodies degradation	R_BDHm	3-Hydroxybutyric acid[m] + NAD^+[m] ⇌ **Acetoacetic acid[m]** + H^+[m] + NADH[m]
		Ketogenesis, leucine degradation	R_HMGLm	3-Hydroxy-3-methylglutaryl-CoA[m] → **Acetoacetic acid[m]** + Acetyl-CoA[m]
M_glu_L_m	L-Glutamate [mitochondrial]	GABA shunt	R_ABTArm	4-aminobutanoate[m] + 2-oxoglutarate[m] ⇌ **L-Glutamate[m]**+ Succinate Semialdehyde[m]
		Beta-alanine degradation	R_APAT2rm	2-oxoglutarate[m] + Beta-alanine[m] ⇌ **L-Glutamate[m]** + 3-Oxopropanoate[m]
		Malate asparte shuttle	ASPGLUmB_MitoCore	L-aspartate[m] + L-glutamate[c] + H^+[c] → L-aspartate[c] + **L-Glutamate[m]** + H^+[m]
		Malate aspartate shuttle	R_ASPTAm	L-Aspartate[m] + 2-Oxoglutarate[m] ⇌ Oxaloacetate[m] + **L-Glutamate[m]**
		Cysteine degradation	R_CYSTAm	2-Oxoglutarate[m] + L-Cysteine[m] ⇌ **L-Glutamate[m]** + Mercaptopyruvate[m]

		Mitochondrial transporters	R_GLUt2mB_MitoCore	L-Glutamate[c] + H$^+$[c]] ⇌ **L-Glutamate[m]** + H$^+$[m]
		Isoleucine degradation	R_ILETAm	2-Oxoglutarate[m] + L-isoleucine[m] ⇌ **L-Glutamate[m]** + 3-Methyl-2-oxopentanoicacid[m]
		Leucine degradation	R_LEUTAm	2-Oxoglutarate[m] + L-Leucine[m] ⇌ **L-Glutamate[m]** + 4-Methyl-2-oxopentanoate[m]
		Ornithine degradation	R_ORNTArm	2-Oxoglutarate[m] + L-Ornithine[m] ⇌ **L-Glutamate[m]** + L-Glutamate 5-semialdehyde[m]
		Valine degradation	R_VALTAm	2-Oxoglutarate[m] + L-Valine[m] ⇌ **L-Glutamate[m]** + 3-Methyl-2-oxobutanoicacid[m]
		Proline, ornithine degradation	R_r0074	L-Glutamate 5-semialdehyde[m] + H2O[m] + NAD+[m] ⇌ **L-Glutamate[m]** + 2 H$^+$[m] + NADH[m]
		Tricarboxylic acid cycle periphery	R_r0081	2-Oxoglutarate[m] + L-Alanine[m] ⇌ **L-Glutamate[m]**+ Pyruvate[m]
		Lysine degradation	R_r0450	2-Oxoglutarate[m] + L-2-Aminoadipate[m] ⇌ **L-Glutamate[m]** + 2-Oxodipate[m]
		Lysine degradation	R_r0525	H2O[m] + NAD+[m] + Saccharopine[m] ⇌ **L-Glutamate[m]** + H$^+$[m] + Allysine[m] + NADH[m]
M_bhb_m	3-Hydroxybutyric acid [mitochondrial]	Mitochondrial transporters	R_BHBtmB_MitoCore	3-Hydroxybutyric acid[c] + H$^+$[c] ⇌ **3-Hydroxybutyric acid[m]** + H$^+$[m]
