# Supplementary material for: Hybrid computational modeling highlights reverse warburg effect in breast cancer-associated fibroblasts
Source: Comput Struct Biotechnol J. 2023 Aug 20;21:4196–206. doi: 10.1016/j.csbj.2023.08.015 (PMC10495551; doi:10.1016/j.csbj.2023.08.015)
Supplement: Supplementary file 6 — Supplementary material [file mmc6.pdf]

Table S6. Metabolic flux distribution in control conditions with maximal ATP production as objective function.

Reaction	Flux
EX_2hb_e	0.0
EX_ac_e	0.0
EX_acac_e	-0.114
EX_akg_e	9.8879238130678e-17
EX_ala_B_e	0.0
EX_ala_L_e	-0.01
EX_arg_L_e	-0.007000000000000002
EX_argsuc_e	0.0
EX_asn_L_e	-0.01
EX_asp_L_e	-0.154
EX_bhb_e	-0.048
EX_bilirub_e	0.0
EX_biomass_e	1.6043502174915354e-14
EX_but_e	-0.006
EX_chol_e	-3.897646500142139e-16
EX_cit_e	0.0
EX_citr_L_e	0.0
EX_co_e	0.0
EX_co2_e	18.306913043478332
EX_creat_e	0.0
EX_cyan_e	-0.001
EX_cys_L_e	-0.001
EX_etoh_e	0.0
EX_fe2_e	0.0
EX_for_e	0.0
EX_fum_e	0.0
EX_glc_D_e	-0.9
EX_gln_L_e	0.0
EX_glu_L_e	0.0
EX_gly_e	-0.009
EX_glyc_e	-0.01
EX_h_e	-3.458111736090018
EX_h2o_e	16.69917391304352
EX_HC00250_e	0.0
EX_hco3_e	-1.71
EX_hdca_e	-0.46836956521739564
EX_his_L_e	-0.01
EX_icit_e	0.0
EX_ile_L_e	-0.004
EX_lac_L_e	-0.575
EX_leu_L_e	-0.016
EX_lys_L_e	-0.03
EX_mal_L_e	9.550954532086937e-15
EX_mercplac_e	0.0

EX_met_L_e	0.0
EX_nad_e	0.0
EX_nadh_e	0.0
EX_nh4_e	0.3619999999999989
EX_no_e	0.0
EX_o2_e	-19.8
EX_oaa_e	0.0
EX_pchol_hs_e	8.228364833633404e-16
EX_pcreat_e	0.0
EX_pe_hs_e	-1.3371339170087263e-14
EX_phe_L_e	0.0
EX_pi_e	-1.787459069646502e-14
EX_ppa_e	0.0
EX_pro_L_e	-0.004
EX_ps_hs_e	-1.1798240444194644e-14
EX_ser_L_e	-0.017
EX_so3_e	0.0
EX_succ_e	0.0
EX_tcynt_e	0.001
EX_thr_L_e	-0.012
EX_trp_L_e	5.631981477367706e-16
EX_tsul_e	0.0
EX_tyr_L_e	0.0
EX_urea_e	0.007000000000000002
EX_val_L_e	-0.011
EX_fol_e	0.0
OF_ATP_MitoCore	0.0
OF_HEME_MitoCore	0.0
OF_LIPID_MitoCore	0.0
OF_PROTEIN_MitoCore	0.0
HEX1	0.9
G6PPer	0.0
PGI	0.9
PFK	0.9
FBP	0.0
FBA	0.9
TPI	0.9099999999999987
GAPD	1.8100000000000003
PGK	1.8100000000000003
PGM	1.8090000000000002
ENO	1.8090000000000002
PYK	3.687999999999999
r0122	0.0
PEPCK	1.7789999999999995
LDH_L	-0.575
G6PDH2r	0.0
PGL	0.0
GND	0.0

RPI	0.0
RPE	0.0
TKT1	0.0
TALA	0.0
TKT2	0.0
PDHm	2.6070000000000015
CSm	6.801956521739163
ACONTm	6.901956521739163
ICDHxm	6.87258543630286
ICDHyrm	0.029371085436303457
AKGDm	6.6389565217391695
SUCOAS1m	-6.48795652173917
SUCOASm	0.0
FUMm	6.849956521739169
MDHm	9.245956521739156
CI_MitoCore	29.371085436302955
CII_MitoCore	6.849956521739169
CIII_MitoCore	39.54125782912759
CIV_MitoCore	19.770628914563794
CV_MitoCore	130.1196537954805
PEPCKm	0.09999999999999777
PCm	1.667
ME2m	0.0
ME1m	0.0
r0081	0.0
ACITLm_MitoCore	0.0
NDPK1m	-6.387956521739172
NNT_MitoCore	0.0
ADK1m	67.88493559339243
ME2	0.0
ALATA_L	0.009999999999999436
NDPK1	1.7789999999999995
FUM	0.0
ADK1	0.46836956521739564
ICDHy	-0.1
ACONT	-0.1
ACITL	0.0
ASPTA	4.174999999999997
MDH	-2.3959999999999977
AKGMALtm	4.284999999999995
ASPGLUmB_MitoCore	4.010999999999997
ASPTAm	-4.010999999999997
G3PD1	0.010000000000000078
r0205	0.0
FACOAL160i	0.46836956521739564
C160CPT1	0.46836956521739564
PPA	0.46836956521739564
r2435	0.46836956521739564

C160CPT2	0.46836956521739564
PPAm	67.88493559339243
ACOT2_MitoCore	0.0
ACADLC16_MitoCore	0.46836956521739564
MECR16C_MitoCore	0.0
MTPC16_MitoCore	0.46836956521739564
ACADLC14_MitoCore	0.46836956521739564
MECR14C_MitoCore	0.0
MTPC14_MitoCore	0.46836956521739564
r1447	0.46836956521739564
r0638	0.0
r0660	-0.46836956521739564
r0722	0.46836956521739564
r0724	-0.46836956521739564
r1451	0.46836956521739564
r0735	0.0
r0728	-0.46836956521739564
r0726	0.46836956521739564
r0634	-0.46836956521739564
r1448	0.46836956521739564
r0633	0.0
r0731	0.46836956521739564
r0730	0.46836956521739564
r0732	0.46836956521739564
r1450	0.46836956521739564
r0791	0.0
r0734	0.46836956521739564
r0733	0.46836956521739564
r0287	0.46836956521739564
r1446	0.47436956521739565
ECOAH1m	-0.5043695652173951
HACD1m	-0.5043695652173951
ACACT1rm	-0.6823695652173951
ACCOAC	0.0
MCOATA	0.0
ACOATA	0.0
r0678	0.0
r0691	0.0
r0681	0.0
r0682	0.0
r0760	0.0
r0761	0.0
r0762	0.0
r0763	0.0
r0764	0.0
r0694	0.0
r0695	0.0
r0765	0.0

r0766	0.0
r0692	0.0
r0693	0.0
r0767	0.0
r0768	0.0
r0769	0.0
r0770	0.0
r0712	0.0
r0713	0.0
r0701	0.0
r0702	0.0
r0771	0.0
r0772	0.0
r0696	0.0
r0697	0.0
r0773	0.0
FA160ACPH	0.0
FACOAL40im	0.006
BDHm	0.048
OCOAT1m	0.178
HMGCOASim	0.0
HMGLm	0.016
LEUTAm	0.016
OIVD1m	0.016
r0655	0.016
MCCCrm	0.016
MGCHrm	0.016
ILETAm	0.004
OIVD3m	0.004
r0603	0.004
ECOAH9m	0.004
HACD9m	0.004
ACACT10m	0.004
VALTAm	0.011
OIVD2m	0.011
r0560	0.011
ECOAH12m	0.011
3HBCOAHLm	0.011
HIBDm	0.011
ACCOALm	67.87893559339243
MMSAD1m	0.011
PPCOACm	0.027
MMEm	-0.027
MMMm	0.027
MMCDm	0.0
RE2649M	67.87893559339243
THRD_L	0.012
r1155	0.012

r1154	0.012
2HBO	0.0
METAT	0.0
METAT2_MitoCore	0.0
AHC	0.0
ADNK1	0.0
CYSTS	0.0
CYSTGL	0.0
CYSO	0.0
3SALATAi	0.0
3SPYRSP	0.0
CYSTA	0.001
CYSTAm	0.0
MCPST	0.001
MCPSTm_MitoCore	0.0
r0595m_MitoCore	0.0
r0595B_MitoCore	0.0
MCLOR	0.0
r0193	0.0
TRPO2	-5.631981477367706e-16
FKYNH	-5.631981477367706e-16
KYN3OX	-5.631981477367706e-16
HKYNH	0.0
3HAO	-5.631981477367706e-16
PCLAD	-5.631981477367706e-16
r0645	-5.631981477367706e-16
AMCOXO	0.0
AMCOXO2_MitoCore	-5.631981477367706e-16
2OXOADPTmB_MitoCore	0.0
2OXOADPTmC_MitoCore	0.0
2OXOADOXm	0.029999999999999437
r0541	0.029999999999999437
SACCD3m	0.03
r0525	0.03
AASAD3m	0.0
R03103_MitoCore	0.03
r0450	0.03
LYSOXc_MitoCore	0.0
PPD2CSPc_MitoCore	0.0
1PPDCRc_MitoCore	0.0
1PPDCRc_NADPH_MitoCore	0.0
LPCOXc_MitoCore	0.0
RE1254C	0.0
r0594	0.0
2AMADPTmB_MitoCore	0.0
2AMADPTmC_MitoCore	0.0
PROD2mB_MitoCore	0.004000000000000001
G5SADrm	-0.004

r0074	0.010999999999999994
GLU5Km	0.0
G5SDym	-5.984795992119984e-17
P5CRm	0.0
P5CRxm	0.0
ORNTArm	0.006999999999999845
ORNDC	0.0
PTRCOX1	0.0
r0464c_MitoCore	0.0
ABUTD	0.0
ARGDCm	0.0
AGMTm	0.0
PTRCAT1m_MitoCore	0.0
APRTO2m_MitoCore	0.0
NABTNOm	0.0
4aabutn_MitoCore	0.0
GLUDC	0.18400000000000025
4ABUTtm	0.18400000000000025
ABTArm	0.18400000000000025
r0178	0.18400000000000025
GLUDxm	0.29300000000000415
GLUDym	0.0
GLUDxi	0.0
GLUDy	0.0
GLNS	-6.661338147750939e-16
GLUNm	0.0
GLUN_MitoCore	0.0
PGCD	0.0010000000000001416
PSERT	0.0009999999999998621
PSP_L	0.0010000000000001416
GHMT2r	0.04499999999999972
FOLR2	0.0
DHFR	0.0
MTHFD	0.04499999999999972
MTHFC	0.05499999999999716
FTCD	0.01
FTHFL	0.0
FTHFDH	0.05499999999999716
r0060	0.0
GHMT2rm	-0.02699999999999986
GCCam	0.02699999999999986
GCCbim	0.02699999999999986
GCCcm	0.02699999999999986
r0514	0.0
r0226	0.0
MTHFDm	0.0
MTHFD2m	0.0
MTHFCm	0.0

FTHFLm	-5.631981477367706e-16
FTHFDHm_MitoCore	0.0
GLYATm	0.0
AOBUTDsm	0.0
AACTOORm_MitoCore	0.0
LGTHLm_MitoCore	0.0
GLYOXm	0.0
LDH_Dm_MitoCore	0.0
CBPSam	1.734723475976807e-18
OCBTm	1.734723475976807e-18
NOS1	0.0
NOS2	0.0
r0129	0.0
AMPTASECG	0.0
GLUCYS	0.0
GTHS	0.0
r0399	0.0
DHPR	0.0
TYRTA	0.0
TYRTB_MitoCore	0.0
34HPPOR	0.0
HGNTOR	0.0
MACACI	0.0
FUMAC	0.0
ASNS1	0.0
r0127	0.01
HISD	0.01
URCN	0.01
IZPN	0.01
GluForTx	0.01
APAT2rm	0.0
MMSAD3m	0.0
MMSAD3m2_MitoCore	0.0
ASP1DC	0.0
CKc	0.0
CK	0.0
ACOAHi	0.0
ALCD2yf	0.0
ALCD2if	0.0
ACALDtm	0.0
ALDD2xm	0.0
ALDD2x	0.0
ACSm	0.0
ACS	0.0
ADSL1	0.0
ADSS	0.0
AMPD1	0.0
ARGN	0.007000000000000002

ARGSL	0.0
ARGSS	0.0
ARGNm	0.0
ALASm	0.0
5AOPtm	0.0
PPBNGS	0.0
HMBS	0.0
UPP3S	0.0
UPPDC1	0.0
CPPPGO	0.0
PPPGOmB_MitoCore	0.0
FCLTm	0.0
PHEMEtm	0.0
HOXG	0.0
BILIRED	0.0
BILIRED2_MitoCore	0.0
PCHOLPm_hs	-3.897646500142139e-16
GLYK	0.01000000000000708
GLYC3Ptm	6.884061941509679e-15
GPAMm_hsB_MitoCore	0.0
AGPAT1B_MitoCore	0.0
CDSm	-3.897646500142139e-16
PGPPTm	6.884061941509679e-15
PGPP_hsm_MitoCore	6.884061941509679e-15
CLS_hsm_MitoCore	0.0
CLPN_MitoCore	7.078944266516786e-15
CYTK1m	6.884061941509679e-15
NDPK3m	6.884061941509679e-15
SPODMm	0.029371085436302957
GTHP	0.0
GTHPm	0.029371085436302957
GTHO	0.0
GTHOm	0.029371085436302957
CITtamB	0.0
r0913	0.0
CITtbm	-0.09999999999999777
r0917	0.0
r0917b_MitoCore	0.0
PIt2mB_MitoCore	-2.7612608695651746
ATPtmB_MitoCore	-0.9722608695651751
HtmB_MitoCore	0.0
MALtm	-1.8890000000000073
MALSO3tm	0.0
MALTSULtm	0.0
MALSO4tm	0.0
SUCCt2m	0.0
r0830	0.0
r0830B_MitoCore	0.0

r0829	0.0
SUCCt3m_MitoCore	0.0
COAtmB_MitoCore	0.0
COAtmC_MitoCore	0.0
GLUt2mB_MitoCore	0.0
ORNt4mB_MitoCore	0.0
r2398B_MitoCore	0.0
r2402B_MitoCore	0.0
LYStmB_MitoCore	0.03
ORNt3mB_MitoCore	-0.007000000000000003
ARGtmB_MitoCore	0.0
r1427	0.0
PYRt2m	4.273999999999998
ACACt2mB_MitoCore	0.114
FE2tm	0.0
ASNtm	0.0
r1437	0.0
GLNtm	-6.661338147750939e-16
PROtm	0.004
r1078	0.0
r1436	0.0
r1455	0.0
TRPtm_MitoCore	0.0
GLYtm	0.05399999999999972
ILEt5m	0.004
LEUt5m	0.016
VALt5m	0.011
r1434	0.0
r1435	-0.026999999999999986
r1440	0.0
BALAtmr	0.0
UREAtm	0.0
FUMtmB_MitoCore	0.0
BHBtmB_MitoCore	0.048
PPAtmB_MitoCore	0.0
BUTt2mB_MitoCore	0.006
FORt2mB_MitoCore	5.631981477367706e-16
r0962B_MitoCore	0.0
CHLtmB_MitoCore	3.897646500142139e-16
CO2tm	-16.388913043478333
H2Otm	-15.461543478260937
O2tm	19.800000000000097
GLYCtm	-7.078944266516786e-15
CYANtm	0.0
TCYNTtmB_MitoCore	0.0
CREATtmdiffir	0.0
PCREATtmdiffirB_MitoCore	0.0
r0941	1.71

r0838B_MitoCore	-0.3199999999999924
Biomasst_MitoCore	0.0
PCFLOPm	-8.228364833633404e-16
PSFLIPm	1.1798240444194644e-14
PEFLIPm	1.3371339170087263e-14
Biomass_MitoCore	0.0
O2t	19.8
CO2t	-18.306913043478332
HCO3t_MitoCore	1.71
GLCt1r	0.9
HDCAtr	0.46836956521739564
HDCAtm_MitoCore	0.0
L_LACt2r	0.575
BHBt	0.048
ACACt2	0.114
ETOHt	0.0
BUTt2r	0.006
GLYCt	-0.01
r0942	0.0
r0942b_MitoCore	0.0
HIStiDF	0.01
ILEtec	0.004
LEUtec	0.016
LYStiDF	0.03
METtec	0.0
PHEtec	0.0
r2534	0.012
TRPt	-5.631981477367706e-16
VALtec	0.011
ARGtiDF	0.007
ASPte	-0.154
CYStec	0.001
GLUt_MitoCore	0.0
r2525	0.0
GLYt2r	0.009
PROt2r	0.004
r2526	0.017
TYRt	0.0
r2532	0.01
ALAt2r	0.01
FUMt_MitoCore	0.0
SUMt_MitoCore	0.0
r0817	0.0
NH4t3r	0.3619999999999989
ACt2r	0.0
PPAt	0.0
2HBt2	0.0
CHOLtu	3.897646500142139e-16

r1088	0.0
ICITt_MitoCore	0.0
UREAt	-0.007000000000000002
r1512	0.0
ARGSUCt_MitoCore	0.0
MAL_Lte	0.0
OAAt_MitoCore	0.0
AKGt_MitoCore	0.0
MERCPLACt_MitoCore	0.0
r0899	0.0
FE2t	0.0
H2Ot	-16.69917391304352
Hct_MitoCore	6.1243695652174095
Hmt_MitoCore	-3.7942578291273903
SO3t_MitoCore	0.0
TSULt_MitoCore	0.0
r0940	0.0
CYANt	0.001
TCYNTt	0.001
r1423	-1.787459069646502e-14
FORt_MitoCore	0.0
FOLt_MitoCore	0.0
NADHt_MitoCore	0.0
NADt_MitoCore	0.0
NADHtm_MitoCore	0.0
NADtm_MitoCore	0.0
COt	0.0
NOt	0.0
PCHOLHSTDe	8.228364833633404e-16
PSt3	1.1798240444194644e-14
PEt	1.3371339170087263e-14
