# Supplementary material for: Hybrid computational modeling highlights reverse warburg effect in breast cancer-associated fibroblasts
Source: Comput Struct Biotechnol J. 2023 Aug 20;21:4196–206. doi: 10.1016/j.csbj.2023.08.015 (PMC10495551; doi:10.1016/j.csbj.2023.08.015)
Supplement: Supplementary file 7 — Supplementary material [file mmc7.pdf]

Table S7. Metabolic flux distribution in breast CAF conditions with maximal ATP production as objective function.

Reaction	Flux
EX_2hb_e	0,01
EX_ac_e	0,01
EX_acac_e	0
EX_akg_e	0
EX_ala_B_e	0
EX_ala_L_e	-0,01
EX_arg_L_e	0
EX_argsuc_e	0
EX_asn_L_e	-0,01
EX_asp_L_e	-0,154
EX_bhb_e	0
EX_bilirub_e	0
EX_biomass_e	0
EX_but_e	-7,98E-17
EX_chol_e	-2,15E-16
EX_cit_e	0
EX_citr_L_e	0
EX_co_e	0
EX_co2_e	0,41
EX_creat_e	0
EX_cyan_e	0
EX_cys_L_e	-0,001
EX_etoh_e	0
EX_fe2_e	0
EX_for_e	0
EX_fum_e	0
EX_glc_D_e	-0,9
EX_gln_L_e	0
EX_glu_L_e	0
EX_gly_e	-3,42E-17
EX_glyc_e	-0,01
EX_h_e	1,402
EX_h2o_e	0
EX_HC00250_e	0
EX_hco3_e	-0,236
EX_hdca_e	1,61E-17
EX_his_L_e	-0,01
EX_icit_e	0
EX_ile_L_e	0
EX_lac_L_e	1,995
EX_leu_L_e	0
EX_lys_L_e	6,16E-33
EX_mal_L_e	0
EX_mercplac_e	0
EX_met_L_e	0
EX_nad_e	0
EX_nadh_e	0
EX_nh4_e	0,225
EX_no_e	0

EX_o2_e	-0,001
EX_oaa_e	1,28E-17
EX_pchol_hs_e	1,47E-16
EX_pcreat_e	0
EX_pe_hs_e	9,37E-17
EX_phe_L_e	0
EX_pi_e	1,91E-17
EX_ppa_e	0
EX_pro_L_e	-1,25E-18
EX_ps_hs_e	7,98E-17
EX_ser_L_e	4,07E-17
EX_so3_e	0,001
EX_succ_e	0
EX_tcynt_e	0
EX_thr_L_e	-0,01
EX_trp_L_e	0
EX_tsul_e	0
EX_tyr_L_e	8,13E-20
EX_urea_e	0
EX_val_L_e	0
EX_fol_e	-1,54E-17
OF_ATP_MitoCore	0
OF_HEME_MitoCore	0
OF_LIPID_MitoCore	0
OF_PROTEIN_MitoCore	0
HEX1	0,9
G6PPer	0
PGI	0,9
PFK	0,9
FBP	0
FBA	0,9
TPI	0,91
GAPD	1,81
PGK	1,81
PGM	1,81
ENO	1,81
PYK	1,984
r0122	0
PEPCK	0,174
LDH_L	1,995
G6PDH2r	0
PGL	0
GND	0
RPI	0
RPE	0
TKT1	0
TALA	0
TKT2	0
PDHm	0
CSm	0
ACONTm	0
ICDHxm	0
ICDHyrm	0

AKGDm	0
SUCOAS1m	0
SUCOASm	0
FUMm	0
MDHm	0
CI_MitoCore	0
CII_MitoCore	0
CIII_MitoCore	0
CIV_MitoCore	0
CV_MitoCore	0,666666667
PEPCKm	0
PCm	0
ME2m	0
ME1m	0
r0081	0
ACITLm_MitoCore	0
NDPK1m	0
NNT_MitoCore	0
ADK1m	1,115333333
ME2	0
ALATA_L	0,01
NDPK1	0,174
FUM	0
ADK1	8,55E-17
ICDHy	-0,01
ACONT	-0,01
ACITL	0,01
ASPTA	0,164
MDH	0
AKGMALtm	0
ASPGLUmB_MitoCore	0
ASPTAm	0
G3PD1	0,01
r0205	0
FACOAL160i	8,55E-17
C160CPT1	8,55E-17
PPA	8,55E-17
r2435	8,55E-17
C160CPT2	8,55E-17
PPAm	1,115333333
ACOT2_MitoCore	0
ACADLC16_MitoCore	0
MECR16C_MitoCore	-1,54E-17
MTPC16_MitoCore	1,54E-17
ACADLC14_MitoCore	0
MECR14C_MitoCore	-1,54E-17
MTPC14_MitoCore	1,54E-17
r1447	1,54E-17
r0638	0
r0660	-1,54E-17
r0722	1,54E-17
r0724	-1,54E-17
r1451	1,54E-17

r0735	0
r0728	7,96E-18
r0726	-7,96E-18
r0634	7,96E-18
r1448	-7,96E-18
r0633	0
r0731	4,15E-17
r0730	4,15E-17
r0732	4,15E-17
r1450	4,15E-17
r0791	0
r0734	4,15E-17
r0733	4,15E-17
r0287	-7,98E-17
r1446	0
ECOAH1m	0
HACD1m	0
ACACT1rm	0
ACCOAC	0
MCOATA	0
ACOATA	0
r0678	0
r0691	0
r0681	0
r0682	0
r0760	0
r0761	0
r0762	0
r0763	0
r0764	0
r0694	0
r0695	0
r0765	0
r0766	0
r0692	0
r0693	0
r0767	0
r0768	0
r0769	0
r0770	0
r0712	0
r0713	0
r0701	0
r0702	0
r0771	0
r0772	0
r0696	0
r0697	0
r0773	0
FA160ACPH	0
FACOAL40im	7,98E-17
BDHm	0
OCOAT1m	0

HMGCOASim	0
HMGLm	0
LEUTAm	0
OIVD1m	0
r0655	0
MCCCrm	0
MGCHrm	0
ILETAm	0
OIVD3m	0
r0603	0
ECOAH9m	0
HACD9m	0
ACACT10m	0
VALTAm	0
OIVD2m	0
r0560	0
ECOAH12m	0
3HBCOAHLm	0
HIBDm	0
ACCOALm	1,115333333
MMSAD1m	0
PPCOACm	0,236
MMEm	0
MMMm	0
MMCDm	0,236
RE2649M	1,115333333
THRD_L	0,01
r1155	0
r1154	0
2HBO	-0,01
METAT	0
METAT2_MitoCore	0
AHC	0
ADNK1	0
CYSTS	0
CYSTGL	0
CYSO	0,001
3SALATAi	0,001
3SPYRSP	0,001
CYSTA	0
CYSTAm	0
MCPST	0
MCPSTm_MitoCore	0
r0595m_MitoCore	0
r0595B_MitoCore	0
MCLOR	0
r0193	0
TRPO2	0
FKYNH	0
KYN3OX	0
HKYNH	0
3HAO	0
PCLAD	0

r0645	0
AMCOXO	0
AMCOXO2_MitoCore	0
2OXOADPTmB_MitoCore	0
2OXOADPTmC_MitoCore	0
2OXOADOXm	0
r0541	0
SACCD3m	0
r0525	0
AASAD3m	0
R03103_MitoCore	0
r0450	0
LYSOXc_MitoCore	-6,16E-33
PPD2CSPc_MitoCore	0
1PPDCRc_MitoCore	0
1PPDCRc_NADPH_MitoCore	0
LPCOXc_MitoCore	0
RE1254C	0
r0594	0
2AMADPTmB_MitoCore	0
2AMADPTmC_MitoCore	0
PROD2mB_MitoCore	-1,54E-17
G5SADrm	0
r0074	0
GLU5Km	0
G5SDym	0
P5CRm	0
P5CRxm	-1,67E-17
ORNTArm	0
ORNDC	0
PTRCOX1	0
r0464c_MitoCore	0
ABUTD	0
ARGDCm	0
AGMTm	0
PTRCAT1m_MitoCore	0
APRTO2m_MitoCore	0
NABTNOm	0
4aabutn_MitoCore	0
GLUDC	0
4ABUTtm	0
ABTArm	0
r0178	0
GLUDxm	0
GLUDym	0
GLUDxi	0,185
GLUDy	0
GLNS	0
GLUNm	0
GLUN_MitoCore	0
PGCD	0
PSERT	0
PSP_L	0

GHMT2r	-6,94E-18
FOLR2	0
DHFR	0
MTHFD	-6,94E-18
MTHFC	0,01
FTCD	0,01
FTHFL	0
FTHFDH	0,01
r0060	0
GHMT2rm	-4,12E-17
GCCam	1,84E-17
GCCbim	1,60E-17
GCCcm	1,60E-17
r0514	1,54E-17
r0226	1,54E-17
MTHFDm	0
MTHFD2m	-5,85E-17
MTHFCm	3,47E-18
FTHFLm	0
FTHFDHm_MitoCore	0
GLYATm	0
AOBUTDsm	0
AACTOORm_MitoCore	0
LGTHLm_MitoCore	0
GLYOXm	0
LDH_Dm_MitoCore	0
CBPSam	0
OCBTm	0
NOS1	0
NOS2	0
r0129	0
AMPTASECG	0
GLUCYS	0
GTHS	0
r0399	0
DHPR	0
TYRTA	0
TYRTB_MitoCore	0
34HPPOR	0
HGNTOR	0
MACACI	-2,71E-20
FUMAC	-2,71E-20
ASNS1	0
r0127	0,01
HISD	0,01
URCN	0,01
IZPN	0,01
GluForTx	0,01
APAT2rm	0
MMSAD3m	0
MMSAD3m2_MitoCore	0
ASP1DC	0
CKc	0

CK	0
ACOAHi	0,01
ALCD2yf	0
ALCD2if	0
ACALDtm	0
ALDD2xm	0
ALDD2x	0
ACSm	0
ACS	0
ADSL1	0
ADSS	0
AMPD1	0
ARGN	0
ARGSL	0
ARGSS	0
ARGNm	0
ALASm	0
5AOPtm	0
PPBNGS	0
HMBS	0
UPP3S	0
UPPDC1	0
CPPPGO	0
PPPGOmB_MitoCore	0
FCLTm	0
PHEMEtm	0
HOXG	0
BILIRED	0
BILIRED2_MitoCore	0
PCHOLPm_hs	-2,15E-16
GLYK	0,01
GLYC3Ptm	-9,71E-17
GPAMm_hsB_MitoCore	0
AGPAT1B_MitoCore	0
CDSm	-9,71E-17
PGPPTm	-9,71E-17
PGPP_hsm_MitoCore	-9,71E-17
CLS_hsm_MitoCore	0
CLPN_MitoCore	-4,86E-17
CYTK1m	-9,71E-17
NDPK3m	-9,71E-17
SPODMm	0
GTHP	0
GTHPm	0
GTHO	-6,16E-33
GTHOm	0
CITtamB	0
r0913	0
CITtbm	0
r0917	0
r0917b_MitoCore	0
PIt2mB_MitoCore	-1,8
ATPtmB_MitoCore	-1,8

HtmB_MitoCore	0
MALtm	0
MALSO3tm	0
MALTSULtm	0
MALSO4tm	0
SUCCt2m	0
r0830	0
r0830B_MitoCore	0
r0829	0
SUCCt3m_MitoCore	0
COAtmB_MitoCore	0
COAtmC_MitoCore	0
GLUt2mB_MitoCore	0
ORNt4mB_MitoCore	0
r2398B_MitoCore	0
r2402B_MitoCore	0
LYStmB_MitoCore	0
ORNt3mB_MitoCore	0
ARGtmB_MitoCore	0
r1427	0
PYRt2m	0
ACACt2mB_MitoCore	0
FE2tm	0
ASNtm	0
r1437	0
GLNtm	0
PROtm	1,25E-18
r1078	0
r1436	0
r1455	0
TRPtm_MitoCore	0
GLYtm	5,00E-17
ILEt5m	0
LEUt5m	0
VALt5m	0
r1434	0
r1435	-3,37E-17
r1440	0
BALAtmr	0
UREAtm	0
FUMtmB_MitoCore	0
BHBtmB_MitoCore	0
PPAtmB_MitoCore	0
BUTt2mB_MitoCore	7,98E-17
FORt2mB_MitoCore	0
r0962B_MitoCore	1,54E-17
CHLtmB_MitoCore	2,15E-16
CO2tm	-0,236
H2Otm	1,564
O2tm	0
GLYCtm	4,86E-17
CYANtm	0
TCYNTtmB_MitoCore	0

CREATtmdiffir	0
PCREATtmdiffirB_MitoCore	0
r0941	0,236
r0838B_MitoCore	-1,84E-17
Biomasst_MitoCore	0
PCFLOPm	-1,47E-16
PSFLIPm	-7,98E-17
PEFLIPm	-9,37E-17
Biomass_MitoCore	0
O2t	0,001
CO2t	-0,41
HCO3t_MitoCore	0,236
GLCt1r	0,9
HDCAtr	-1,61E-17
HDCAtm_MitoCore	0
L_LACt2r	-1,995
BHBt	0
ACACt2	0
ETOHt	0
BUTt2r	7,98E-17
GLYCt	-0,01
r0942	0
r0942b_MitoCore	0
HIStiDF	0,01
ILEtec	0
LEUtec	0
LYStiDF	-6,16E-33
METtec	0
PHEtec	0
r2534	0,01
TRPt	0
VALtec	0
ARGtiDF	0
ASPte	-0,154
CYStec	0,001
GLUt_MitoCore	0
r2525	0
GLYt2r	5,40E-17
PROt2r	1,25E-18
r2526	-4,07E-17
TYRt	-8,13E-20
r2532	0,01
ALAt2r	0,01
FUMt_MitoCore	0
SUMt_MitoCore	0
r0817	0
NH4t3r	0,225
ACt2r	-0,01
PPAt	0
2HBt2	-0,01
CHOLtu	2,15E-16
r1088	0
ICITt_MitoCore	0

UREAt	0
r1512	0
ARGSUCt_MitoCore	0
MAL_Lte	0
OAAt_MitoCore	0
AKGt_MitoCore	0
MERCPLACt_MitoCore	0
r0899	0
FE2t	0
H2Ot	0
Hct_MitoCore	0,142
Hmt_MitoCore	0,236
SO3t_MitoCore	-0,001
TSULt_MitoCore	0
r0940	0
CYANt	0
TCYNTt	0
r1423	-2,73E-16
FORt_MitoCore	0
FOLt_MitoCore	1,54E-17
NADHt_MitoCore	0
NADt_MitoCore	0
NADHtm_MitoCore	0
NADtm_MitoCore	0
COt	0
NOt	0
PCHOLHSTDe	1,47E-16
PSt3	-7,98E-17
PEt	-9,37E-17
