# Supplementary material for: Hybrid computational modeling highlights reverse warburg effect in breast cancer-associated fibroblasts
Source: Comput Struct Biotechnol J. 2023 Aug 20;21:4196–206. doi: 10.1016/j.csbj.2023.08.015 (PMC10495551; doi:10.1016/j.csbj.2023.08.015)
Supplement: Supplementary file 8 — Supplementary material [file mmc8.pdf]

Table S8. Regulatory knock-out/knock-in FBA simulation results in terms of percentage of total cellular ATP production from glycolytic and oxidative pathways. Each component's value initially set to 1/0 in breast CAF-specific conditions was alternatively set to 0/1 while the others remained unchanged (the details of each set of initial conditions is provided in the GitLab). Condition C57 corresponds to the knock-out of HIF1 (other regulatory inputs do not seem to directly affect metabolic fluxes distribution in breast CAFs).

	**GLYC**	**OXPHOS**
C1	85.05	14.95
C2	85.05	14.95
C3	85.05	14.95
C4	85.05	14.95
C5	85.05	14.95
C6	85.05	14.95
C7	85.05	14.95
C8	85.05	14.95
C9	85.05	14.95
C10	85.05	14.95
C11	85.05	14.95
C12	85.05	14.95
C13	85.05	14.95
C14	85.05	14.95
C15	85.05	14.95
C16	85.05	14.95
C17	85.05	14.95
C18	85.05	14.95
C19	85.05	14.95
C20	85.05	14.95
C21	85.05	14.95
C22	85.05	14.95
C23	85.05	14.95
C24	85.05	14.95
C25	85.05	14.95
C26	85.05	14.95
C27	85.05	14.95
C28	85.05	14.95
C29	85.05	14.95
C30	85.05	14.95
C31	85.05	14.95
C32	85.05	14.95
C33	85.05	14.95
C34	85.05	14.95
C35	85.05	14.95
C36	85.05	14.95
C37	85.05	14.95
C38	85.05	14.95
C39	85.05	14.95
C40	85.05	14.95
C41	85.05	14.95
C42	85.05	14.95
C43	85.05	14.95
C44	85.05	14.95
C45	85.05	14.95
C46	85.05	14.95
C47	85.05	14.95
C48	85.05	14.95
C49	85.05	14.95
C50	85.05	14.95
C51	85.05	14.95
C52	85.05	14.95
C53	85.05	14.95
C54	85.05	14.95
C55	100.0	0
C56	85.05	14.95
**C57**	**0.00**	**100**
C58	85.05	14.95
C59	85.05	14.95
C60	85.05	14.95
C61	85.05	14.95
C62	85.05	14.95
C63	85.05	14.95
C64	85.05	14.95
C65	85.05	14.95
C66	85.05	14.95
C67	85.05	14.95
C68	85.05	14.95
C69	85.05	14.95
C70	85.05	14.95
C71	85.05	14.95
C72	85.05	14.95
C73	85.05	14.95
C74	85.05	14.95
C75	85.05	14.95
C76	85.05	14.95
C77	85.05	14.95
C78	85.05	14.95
C79	85.05	14.95
C80	85.05	14.95
C81	85.05	14.95
C82	85.05	14.95
C83	85.05	14.95
C84	85.05	14.95
C85	85.05	14.95
C86	85.05	14.95
C87	85.05	14.95
C88	85.05	14.95
C89	85.05	14.95
C90	85.05	14.95
C91	85.05	14.95
C92	85.05	14.95
C93	85.05	14.95
C94	85.05	14.95
C95	85.05	14.95
C96	85.05	14.95
C97	85.05	14.95
C98	85.05	14.95
C99	85.05	14.95
C100	85.05	14.95
C101	85.05	14.95
C102	85.05	14.95
C103	85.05	14.95
C104	85.05	14.95
C105	85.05	14.95
C106	85.05	14.95
C107	85.05	14.95
C108	85.05	14.95
C109	85.05	14.95
C110	85.05	14.95
C111	85.05	14.95
C112	85.05	14.95
C113	85.05	14.95
C114	85.05	14.95
C115	85.05	14.95
C116	85.05	14.95
C117	85.05	14.95
C118	85.05	14.95
C119	85.05	14.95
C120	85.05	14.95
C121	85.05	14.95
C122	85.05	14.95
C123	85.05	14.95
C124	85.05	14.95
C125	85.05	14.95
C126	85.05	14.95
C127	85.05	14.95
C128	85.05	14.95
C129	85.05	14.95
C130	85.05	14.95
C131	85.05	14.95
C132	85.05	14.95
C133	85.05	14.95
C134	85.05	14.95
C135	85.05	14.95
C136	85.05	14.95
C137	85.05	14.95
C138	85.05	14.95
C139	85.05	14.95
C140	85.05	14.95
C141	85.05	14.95
C142	85.05	14.95
C143	85.05	14.95
C144	85.05	14.95
C145	85.05	14.95
C146	85.05	14.95
C147	85.05	14.95
