# Supplementary material for: Hybrid computational modeling highlights reverse warburg effect in breast cancer-associated fibroblasts
Source: Comput Struct Biotechnol J. 2023 Aug 20;21:4196–206. doi: 10.1016/j.csbj.2023.08.015 (PMC10495551; doi:10.1016/j.csbj.2023.08.015)
Supplement: Supplementary file 9 — Supplementary material [file mmc9.pdf]

Table S9. Metabolic flux distribution in RASF-specific conditions with maximal ATP production as objective function.

Reaction	Flux
EX_2hb_e	0.0
EX_ac_e	0.01
EX_acac_e	0.0
EX_akg_e	0.0
EX_ala_B_e	0.0
EX_ala_L_e	0.16399999999991904
EX_arg_L_e	-4.1154826743038073e-16
EX_argsuc_e	0.0
EX_asn_L_e	-0.01
EX_asp_L_e	-0.154
EX_bhb_e	0.0
EX_bilirub_e	0.0
EX_biomass_e	0.0
EX_but_e	2.958017715363895e-16
EX_chol_e	9.713566402487743e-20
EX_cit_e	0.0
EX_citr_L_e	0.0
EX_co_e	0.0
EX_co2_e	0.22400000000003512
EX_creat_e	0.0
EX_cyan_e	0.0
EX_cys_L_e	0.0
EX_etoh_e	0.0
EX_fe2_e	0.0
EX_for_e	0.0
EX_fum_e	0.0
EX_glc_D_e	-0.9
EX_gln_L_e	0.0
EX_glu_L_e	0.0
EX_gly_e	-0.005000000000004146
EX_glyc_e	-0.01
EX_h_e	1.5809999999999393
EX_h2o_e	0.0
EX_HC00250_e	0.0
EX_hco3_e	-0.055000000000079284
EX_hdca_e	-6.382746562357108e-17
EX_his_L_e	-0.01
EX_icit_e	0.0
EX_ile_L_e	0.0
EX_lac_L_e	1.8200000000001115
EX_leu_L_e	0.0
EX_lys_L_e	0.0
EX_mal_L_e	0.0
EX_mercplac_e	0.0
EX_met_L_e	0.0

EX_nad_e	0.0
EX_nadh_e	0.0
EX_nh4_e	0.04000000000007814
EX_no_e	0.0
EX_o2_e	0.0
EX_oaa_e	0.0
EX_pchol_hs_e	1.665504612139037e-15
EX_pcreat_e	0.0
EX_pe_hs_e	7.925823692853511e-18
EX_phe_L_e	4.6407322429331543e-14
EX_pi_e	-2.6374234983830386e-15
EX_ppa_e	-2.220446049250313e-16
EX_pro_L_e	-7.322435054995155e-16
EX_ps_hs_e	6.99337384663545e-18
EX_ser_L_e	0.005000000000040563
EX_so3_e	0.0
EX_succ_e	0.0
EX_tcynt_e	0.0
EX_thr_L_e	0.0
EX_trp_L_e	2.8553012288735373e-16
EX_tsul_e	0.0
EX_tyr_L_e	-4.6407322429331543e-14
EX_urea_e	0.0
EX_val_L_e	0.0
EX_fol_e	4.536193960895619e-17
OF_ATP_MitoCore	-1.4882007175434233e-16
OF_HEME_MitoCore	0.0
OF_LIPID_MitoCore	0.0
OF_PROTEIN_MitoCore	0.0
HEX1	0.9
G6PPer	0.0
PGI	0.9
PFK	0.9
FBP	0.0
FBA	0.9
TPI	0.9100000000000327
GAPD	1.8100000000000325
PGK	1.8100000000000325
PGM	1.8100000000000325
ENO	1.8100000000000325
PYK	1.9840000000000315
r0122	0.0
PEPCK	0.17399999999999907
LDH_L	1.8200000000001115
G6PDH2r	0.0
PGL	0.0
GND	0.0
RPI	0.0

RPE	0.0
TKT1	0.0
TALA	0.0
TKT2	0.0
PDHm	0.0
CSm	0.0
ACONTm	0.0
ICDHxm	0.0
ICDHyrm	0.0
AKGDm	0.0
SUCOAS1m	0.0
SUCOASm	0.0
FUMm	0.0
MDHm	0.0
CI_MitoCore	0.0
CII_MitoCore	0.0
CIII_MitoCore	0.0
CIV_MitoCore	0.0
CV_MitoCore	0.6666666666666765
PEPCKm	0.0
PCm	0.0
ME2m	0.0
ME1m	0.0
r0081	2.8553012288735373e-16
ACITLm_MitoCore	0.0
NDPK1m	0.0
NNT_MitoCore	0.0
ADK1m	1.2058333333333107
ME2	0.0
ALATA_L	-0.1639999999999196
NDPK1	0.17399999999999907
FUM	0.0
ADK1	0.0
ICDHy	-0.009999999999999459
ACONT	-0.009999999999999459
ACITL	0.009999999999999459
ASPTA	0.16399999999999962
MDH	0.0
AKGMALtm	0.0
ASPGLUmB_MitoCore	0.0
ASPTAm	0.0
G3PD1	0.01000000000003265
r0205	0.0
FACOAL160i	0.0
C160CPT1	0.0
PPA	0.0
r2435	5.0663992349903355e-15
C160CPT2	5.0663992349903355e-15

PPAm	1.20583333333331
ACOT2_MitoCore	3.967294888299232e-18
ACADLC16_MitoCore	0.0
MECR16C_MitoCore	5.993889820329745e-16
MTPC16_MitoCore	-5.993889820329745e-16
ACADLC14_MitoCore	0.0
MECR14C_MitoCore	-1.3469227005491047e-16
MTPC14_MitoCore	1.3469227005491047e-16
r1447	1.3469227005491047e-16
r0638	0.0
r0660	6.345341554735749e-17
r0722	-6.345341554735749e-17
r0724	6.345341554735749e-17
r1451	-6.345341554735749e-17
r0735	0.0
r0728	6.345341554735749e-17
r0726	-6.345341554735749e-17
r0634	6.345341554735749e-17
r1448	-6.345341554735749e-17
r0633	0.0
r0731	2.958017715363895e-16
r0730	2.958017715363895e-16
r0732	2.958017715363895e-16
r1450	2.958017715363895e-16
r0791	0.0
r0734	2.958017715363895e-16
r0733	2.958017715363895e-16
r0287	2.958017715363895e-16
r1446	0.0
ECOAH1m	0.0
HACD1m	0.0
ACACT1rm	0.0
ACCOAC	-4.747269084961008e-16
MCOATA	-4.747269084961009e-16
ACOATA	-6.779476051187031e-17
r0678	-6.779476051187031e-17
r0691	6.779476051187031e-17
r0681	4.9986044744784655e-15
r0682	6.779476051187031e-17
r0760	-6.779476051187031e-17
r0761	6.779476051187031e-17
r0762	4.9986044744784655e-15
r0763	6.779476051187031e-17
r0764	-6.779476051187031e-17
r0694	6.779476051187031e-17
r0695	4.9986044744784655e-15
r0765	6.779476051187031e-17
r0766	-6.779476051187031e-17

r0692	6.779476051187031e-17
r0693	4.9986044744784655e-15
r0767	6.787655296837461e-17
r0768	-6.787655296837461e-17
r0769	6.787655296837461e-17
r0770	4.9986044744784655e-15
r0712	6.787655296837461e-17
r0713	-6.787655296837461e-17
r0701	6.787655296837461e-17
r0702	4.9986044744784655e-15
r0771	6.779476051187031e-17
r0772	-6.779476051187031e-17
r0696	6.779476051187031e-17
r0697	4.9986044744784655e-15
r0773	6.779476051187031e-17
FA160ACPH	-6.779476051187031e-17
FACOAL40im	-2.958017715363895e-16
BDHm	0.0
OCOAT1m	0.0
HMGCOASim	0.0
HMGLm	0.0
LEUTAm	0.0
OIVD1m	0.0
r0655	0.0
MCCCrm	0.0
MGCHrm	0.0
ILETAm	0.0
OIVD3m	0.0
r0603	0.0
ECOAH9m	0.0
HACD9m	0.0
ACACT10m	0.0
VALTAm	0.0
OIVD2m	0.0
r0560	0.0
ECOAH12m	-5.83822147337224e-16
3HBCOAHLm	-5.83822147337224e-16
HIBDm	-5.83822147337224e-16
ACCOALm	1.205833333333311
MMSAD1m	-5.83822147337224e-16
PPCOACm	0.055000000000079756
MMEm	0.0
MMMm	0.0
MMCDm	0.05500000000007742
RE2649M	1.2058333333333109
THRD_L	0.0
r1155	0.0
r1154	0.0

2HBO	0.0
METAT	0.0
METAT2_MitoCore	0.0
AHC	0.0
ADNK1	0.0
CYSTS	0.0
CYSTGL	0.0
CYSO	0.0
3SALATAi	0.0
3SPYRSP	0.0
CYSTA	0.0
CYSTAm	0.0
MCPST	0.0
MCPSTm_MitoCore	0.0
r0595m_MitoCore	0.0
r0595B_MitoCore	0.0
MCLOR	0.0
r0193	0.0
TRPO2	-2.8553012288735373e-16
FKYNH	-2.8553012288735373e-16
KYN3OX	-2.8553012288735373e-16
HKYNH	-2.8553012288735373e-16
3HAO	-2.8553012288735373e-16
PCLAD	-2.8553012288735373e-16
r0645	-2.8553012288735373e-16
AMCOXO	0.0
AMCOXO2_MitoCore	-2.8553012288735373e-16
2OXOADPTmB_MitoCore	-2.8553012288735373e-16
2OXOADPTmC_MitoCore	0.0
2OXOADOXm	0.0
r0541	0.0
SACCD3m	0.0
r0525	1.531968729397165e-16
AASAD3m	0.0
R03103_MitoCore	0.0
r0450	0.0
LYSOXc_MitoCore	-1.531968729397165e-16
PPD2CSPc_MitoCore	-1.531968729397165e-16
1PPDCRc_MitoCore	-1.531968729397165e-16
1PPDCRc_NADPH_MitoCore	0.0
LPCOXc_MitoCore	-1.531968729397165e-16
RE1254C	-1.531968729397165e-16
r0594	-1.531968729397165e-16
2AMADPTmB_MitoCore	-1.531968729397165e-16
2AMADPTmC_MitoCore	0.0
PROD2mB_MitoCore	-1.4754276757969173e-18
G5SADrm	-2.4505100871085655e-16
r0074	0.0

GLU5Km	-1.4017379891161596e-15
G5SDym	-1.4017379891161596e-15
P5CRm	0.0
P5CRxm	-2.465264363866533e-16
ORNTArm	0.0
ORNDC	0.0
PTRCOX1	0.0
r0464c_MitoCore	0.0
ABUTD	0.0
ARGDCm	0.0
AGMTm	0.0
PTRCAT1m_MitoCore	0.0
APRTO2m_MitoCore	0.0
NABTNOm	0.0
4aabutn_MitoCore	0.0
GLUDC	0.0
4ABUTtm	0.0
ABTArm	0.0
r0178	0.0
GLUDxm	0.0
GLUDym	0.0
GLUDxi	0.0
GLUDy	0.010000000000079926
GLNS	0.0
GLUNm	0.0
GLUN_MitoCore	0.0
PGCD	0.0
PSERT	0.0
PSP_L	0.0
GHMT2r	-0.005000000000040611
FOLR2	0.0
DHFR	0.0
MTHFD	-0.005000000000040611
MTHFC	0.004999999999958602
FTCD	0.009999999999999213
FTHFL	0.0
FTHFDH	0.004999999999958602
r0060	0.0
GHMT2rm	-5.3518981308959085e-17
GCCam	6.5596328190013e-16
GCCbim	6.5596328190013e-16
GCCcm	6.5596328190013e-16
r0514	-4.536193960895619e-17
r0226	-4.536193960895619e-17
MTHFDm	0.0
MTHFD2m	6.024443005911709e-16
MTHFCm	2.8553012288735373e-16
FTHFLm	-2.8553012288735373e-16

FTHFDHm_MitoCore	0.0
GLYATm	0.0
AOBUTDsm	0.0
AACTOORm_MitoCore	0.0
LGTHLm_MitoCore	-7.133966538364059e-16
GLYOXm	-7.133966538364059e-16
LDH_Dm_MitoCore	-7.133966538364059e-16
CBPSam	0.0
OCBTm	0.0
NOS1	0.0
NOS2	0.0
r0129	0.0
AMPTASECG	0.0
GLUCYS	0.0
GTHS	0.0
r0399	-4.6407322429331543e-14
DHPR	-4.6407322429331543e-14
TYRTA	0.0
TYRTB_MitoCore	0.0
34HPPOR	0.0
HGNTOR	0.0
MACACI	0.0
FUMAC	0.0
ASNS1	0.0
r0127	0.009999999999999658
HISD	0.009999999999999213
URCN	0.009999999999999213
IZPN	0.009999999999999213
GluForTx	0.009999999999999213
APAT2rm	0.0
MMSAD3m	0.0
MMSAD3m2_MitoCore	0.0
ASP1DC	0.0
CKc	0.0
CK	0.0
ACOAHi	0.01
ALCD2yf	0.0
ALCD2if	0.0
ACALDtm	0.0
ALDD2xm	0.0
ALDD2x	0.0
ACSm	0.0
ACS	0.0
ADSL1	0.0
ADSS	0.0
AMPD1	0.0
ARGN	0.0
ARGSL	0.0

ARGSS	0.0
ARGNm	0.0
ALASm	0.0
5AOPtm	0.0
PPBNGS	0.0
HMBS	0.0
UPP3S	0.0
UPPDC1	0.0
CPPPGO	0.0
PPPGOmB_MitoCore	0.0
FCLTm	0.0
PHEMEtm	0.0
HOXG	0.0
BILIRED	0.0
BILIRED2_MitoCore	0.0
PCHOLPm_hs	-9.067559395949707e-16
GLYK	0.010000000000034016
GLYC3Ptm	1.366820810469779e-15
GPAMm_hsB_MitoCore	0.0
AGPAT1B_MitoCore	3.1840401608148755e-16
CDSm	-1.3377760975953689e-15
PGPPTm	1.0484167943882912e-15
PGPP_hsm_MitoCore	1.0484167943882912e-15
CLS_hsm_MitoCore	0.0
CLPN_MitoCore	7.462530021191767e-16
CYTK1m	1.221569674310888e-15
NDPK3m	1.221569674310888e-15
SPODMm	0.0
GTHP	-3.06393745879433e-16
GTHPm	0.0
GTHO	-3.06393745879433e-16
GTHOm	0.0
CITtamB	0.0
r0913	0.0
CITtbm	0.0
r0917	0.0
r0917b_MitoCore	0.0
PIt2mB_MitoCore	-1.8000000000000236
ATPtmB_MitoCore	-1.8000000000000231
HtmB_MitoCore	0.0
MALtm	0.0
MALSO3tm	0.0
MALTSULtm	0.0
MALSO4tm	0.0
SUCCt2m	0.0
r0830	0.0
r0830B_MitoCore	0.0
r0829	0.0

SUCCt3m_MitoCore	0.0
COAtmB_MitoCore	0.0
COAtmC_MitoCore	0.0
GLUt2mB_MitoCore	0.0
ORNt4mB_MitoCore	0.0
r2398B_MitoCore	0.0
r2402B_MitoCore	0.0
LYStmB_MitoCore	1.531968729397165e-16
ORNt3mB_MitoCore	0.0
ARGtmB_MitoCore	0.0
r1427	0.0
PYRt2m	4.278665309490522e-16
ACACt2mB_MitoCore	0.0
FE2tm	0.0
ASNtm	0.0
r1437	0.0
GLNtm	0.0
PROtm	2.450510087108564e-16
r1078	0.0
r1436	0.0
r1455	0.0
TRPtm_MitoCore	0.0
GLYtm	8.489086307160221e-16
ILEt5m	0.0
LEUt5m	0.0
VALt5m	0.0
r1434	2.8553012288735373e-16
r1435	4.8038496257819257e-17
r1440	0.0
BALAtmr	0.0
UREAtm	0.0
FUMtmB_MitoCore	0.0
BHBtmB_MitoCore	0.0
PPAtmB_MitoCore	2.220446049250313e-16
BUTt2mB_MitoCore	-2.958017715363895e-16
FORt2mB_MitoCore	2.8553012288735373e-16
r0962B_MitoCore	-4.536193960895619e-17
CHLtmB_MitoCore	1.6561801136768564e-15
CO2tm	-0.05500000000007764
H2Otm	1.7449999999999435
O2tm	0.0
GLYCtm	-7.462530021191767e-16
CYANtm	0.0
TCYNTtmB_MitoCore	0.0
CREATtmdiffir	0.0
PCREATtmdiffirB_MitoCore	0.0
r0941	0.055000000000079756
r0838B_MitoCore	-5.71618936124331e-16

Biomasst_MitoCore	-1.4882007175434233e-16
PCFLOPm	-1.665504612139037e-15
PSFLIPm	-6.99337384663545e-18
PEFLIPm	-7.925823692853511e-18
Biomass_MitoCore	0.0
O2t	-4.757030654387304e-14
CO2t	-0.22400000000003512
HCO3t_MitoCore	0.055000000000079284
GLCt1r	0.9
HDCAtr	6.779476051187031e-17
HDCAtm_MitoCore	-3.967294888299232e-18
L_LACt2r	-1.8200000000001115
BHBt	0.0
ACACt2	0.0
ETOHt	0.0
BUTt2r	-2.958017715363895e-16
GLYCt	-0.01
r0942	0.0
r0942b_MitoCore	0.0
HIStiDF	0.01
ILEtec	0.0
LEUtec	0.0
LYStiDF	0.0
METtec	0.0
PHEtec	-4.6407322429331543e-14
r2534	0.0
TRPt	-2.8553012288735373e-16
VALtec	0.0
ARGtiDF	0.0
ASPte	-0.154
CYStec	0.0
GLUt_MitoCore	0.0
r2525	0.0
GLYt2r	0.00500000000004146
PROt2r	7.322435054995155e-16
r2526	-0.005000000000040563
TYRt	4.6407322429331543e-14
r2532	0.01
ALAt2r	-0.16399999999991904
FUMt_MitoCore	0.0
SUMt_MitoCore	0.0
r0817	0.0
NH4t3r	0.04000000000007814
ACt2r	-0.01
PPAt	2.220446049250313e-16
2HBt2	0.0
CHOLtu	1.6561801136768564e-15
r1088	0.0

ICITt_MitoCore	0.0
UREAt	0.0
r1512	0.0
ARGSUCt_MitoCore	0.0
MAL_Lte	0.0
OAAt_MitoCore	0.0
AKGt_MitoCore	0.0
MERCPLACt_MitoCore	0.0
r0899	0.0
FE2t	0.0
H2Ot	0.0
Hct_MitoCore	0.3129999999998878
Hmt_MitoCore	0.055000000000083295
SO3t_MitoCore	0.0
TSULt_MitoCore	0.0
r0940	0.0
CYANt	0.0
TCYNTt	0.0
r1423	-2.6374234983830386e-15
FORt_MitoCore	0.0
FOLt_MitoCore	-4.536193960895619e-17
NADHt_MitoCore	0.0
NADt_MitoCore	0.0
NADHtm_MitoCore	0.0
NADtm_MitoCore	0.0
COt	0.0
NOt	0.0
PCHOLHSTDe	1.665504612139037e-15
PSt3	-6.99337384663545e-18
PEt	-7.925823692853511e-18
